# *meso*-3,6-Dioxopiperazine-2,5-diacet­amide

**DOI:** 10.1107/S1600536811043376

**Published:** 2011-10-29

**Authors:** Ping Li, Chun Zhang, Wei Xu

**Affiliations:** a Center of Applied Solid State Chemistry Research, Ningbo University, Ningbo, Zhejiang 315211, People’s Republic of China

## Abstract

The title compound, C_8_H_12_N_4_O_4_, was obtained by cyclization of the two l-asparagine mol­ecules and reveals a crystallographic inversion symmetry, and accordingly the two stereogenic centres are of opposite chirality. Thus, an asymmetric unit comprises a half of a mol­ecule. The mol­ecules are assembled into a three-dimensional hydrogen-bonding network by N—H⋯O hydrogen bonds.

## Related literature

For general background to coordination polymers, see: Anitha *et al.* (2005 ▶); Aarthy *et al.* (2005 ▶); Guenifa *et al.* (2009 ▶); Moussa Slimane *et al.* (2009 ▶). For related structures, see: Howes *et al.* (1983 ▶).
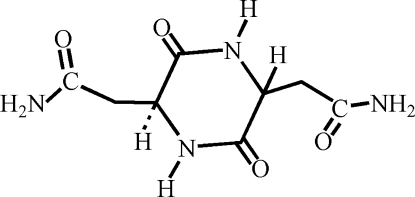


## Experimental

### 

#### Crystal data

C_8_H_12_N_4_O_4_
*M* *_r_* = 228.22
Monoclinic, 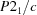

*a* = 5.0409 (10) Å
*b* = 8.3178 (17) Å
*c* = 12.900 (3) Å
β = 109.76 (3)°
*V* = 509.0 (2) Å^3^
*Z* = 2
Mo *K*α radiation
μ = 0.12 mm^−1^
*T* = 293 K
0.10 × 0.10 × 0.10 mm

#### Data collection

Rigaku R-AXIS RAPID diffractometer
Absorption correction: multi-scan (*ABSCOR*; Higashi, 1995 ▶) *T*_min_ = 0.988, *T*_max_ = 0.988
4836 measured reflections
1166 independent reflections
889 reflections with *I* > 2σ(*I*)
*R*_int_ = 0.028

#### Refinement

*R*[*F* ^2^ > 2σ(*F* ^2^)] = 0.037
*wR*(*F* ^2^) = 0.098
*S* = 1.07
1166 reflections
73 parameters
H-atom parameters constrained
Δρ_max_ = 0.24 e Å^−3^
Δρ_min_ = −0.16 e Å^−3^

### 

Data collection: *RAPID-AUTO* (Rigaku, 1998 ▶); cell refinement: *RAPID-AUTO*; data reduction: *CrystalStructure* (Rigaku/MSC, 2004 ▶); program(s) used to solve structure: *SHELXS97* (Sheldrick, 2008 ▶); program(s) used to refine structure: *SHELXL97* (Sheldrick, 2008 ▶); molecular graphics: *SHELXTL* (Sheldrick, 2008 ▶); software used to prepare material for publication: *SHELXL97*.

## Supplementary Material

Crystal structure: contains datablock(s) global, I. DOI: 10.1107/S1600536811043376/kp2350sup1.cif

Structure factors: contains datablock(s) I. DOI: 10.1107/S1600536811043376/kp2350Isup2.hkl

Supplementary material file. DOI: 10.1107/S1600536811043376/kp2350Isup3.cml

Additional supplementary materials:  crystallographic information; 3D view; checkCIF report

## Figures and Tables

**Table 1:** Hydrogen-bond geometry (Å, °)

*D*—H⋯*A*	*D*—H	H⋯*A*	*D*⋯*A*	*D*—H⋯*A*
N1—H1*A*⋯O2^i^	0.86	2.12	2.9185 (19)	154
N1—H1*B*⋯O2^ii^	0.86	2.03	2.8795 (18)	167
N2—H2*C*⋯O1^iii^	0.86	2.06	2.8509 (17)	152

Symmetry codes: (i) 
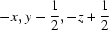
; (ii) 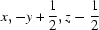; (iii) 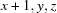.

**Fig. 1.**
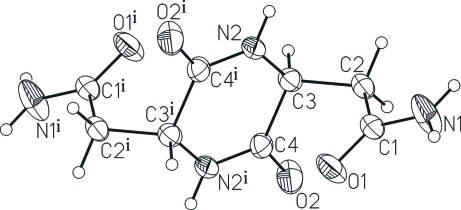
ORTEP view of the title compound. The dispalcement ellipsoids are drawn at 45% probability dispalcement ellipsoids. [Symmetry codes: (i)–x+1, -y, –z+1.]

**Fig. 2.**
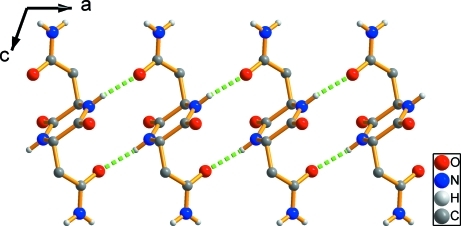
Packing diagram of the title crystal structure viewed down along [010] direction with N2-H2C···O1 hydrogen bond motif.

**Fig. 3.**
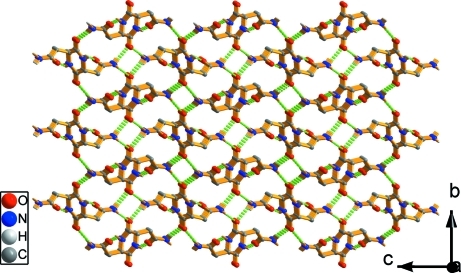
Packing diagram of the title crystal viewed down the a axis shows 3D-hydrogen bond network. N–H···O hydrogen bonds are shown as dashed lines.

## supplementary crystallographic information

### Comment

The past decade has witnessed enormous expansion of research on non-centrosymmetric coordination polymers. For such purpose, rational design and synthesis have been focused on choices of metal cations with non-centrosymmetric organic ligands. Asparagine (Anitha *et al.*, (2005); Aarthy *et al.*, (2005); Guenifa *et al.*, (2009); Moussa Slimane *et al.*, (2009)) is a chiral molecule and one of the common neutral amino acids with carboxamide as the side-chain functional group. However, condensation led to a centrosyymmetric compound and  we report its crystal structure.

In (I) (Fig. 1), two L–asparagine molecules engage in the dehydration condensation between each carboxyl and the adjacent amino groups. The resulting product reveals the molecular symmetry *C*_i_ (crystallographic inversion symmetry). In (I) a piperazinedione-2,5 unit is close to be planar (the mean value of intracyclic torsion angles is 2.65 °) and it is different to those reported by (Howes *et al.*, (1983)). The  molecules are connected through N1–H1A···O2^i^, N1–H1B···O2^ii^, and N2–H2C···O1^iii^  hydrogen bonds generating a  3D-network (Table 1, Figs. 2 and 3.

### Experimental

Dropwise addition of 1 *M* NaOH (1.0 mL) to a stirred aqueous solution of (0.1438 g, 0.5 mmol) ZnSO_4_.7H_2_O in 5.0 mL H_2_O produced pale-white Zn(OH)_2_.xH_2_O precipitate, which was separated by centrifugation and washed with distilled water for several times. Subsequently, the 0.1501 g (1.0 mmol) L–asparagine was dissolved completely with 10.0 mL H_2_O, and then the precipitate was added. The resulting mixture was further stirred at 323 K for 1 h and then filtered. The white filtrate was allowed to stand at room temperature. Slow evaporation for several days afforded colourless needle-like crystals.

### Refinement

H atoms bonded to C atoms were placed in their geometrically calculated positions and refined using the riding model, with C–H distances 0.93Å and *U*_iso_(H) = 1.2 *U*_eq_(C).

### Crystal data

**Table:**

C_8_H_12_N_4_O_4_	*F*(000) = 240
*M**_r_* = 228.22	*D*_x_ = 1.489 Mg m^−^^3^
Monoclinic, *P*2_1_/*c*	Mo *K*α radiation, λ = 0.71073 Å
Hall symbol: -P 2ybc	Cell parameters from 3368 reflections
*a* = 5.0409 (10) Å	θ = 3.4–27.4°
*b* = 8.3178 (17) Å	µ = 0.12 mm^−^^1^
*c* = 12.900 (3) Å	*T* = 293 K
β = 109.76 (3)°	Needle, colourless
*V* = 509.0 (2) Å^3^	0.10 × 0.10 × 0.10 mm
*Z* = 2	

### Data collection

**Table:**

Rigaku R-AXIS RAPID diffractometer	1166 independent reflections
Radiation source: fine-focus sealed tube	889 reflections with *I* > 2σ(*I*)
graphite	*R*_int_ = 0.028
Detector resolution: 0 pixels mm^-1^	θ_max_ = 27.5°, θ_min_ = 3.4°
ω scans	*h* = −6→5
Absorption correction: multi-scan (*ABSCOR*; Higashi, 1995)	*k* = −10→10
*T*_min_ = 0.988, *T*_max_ = 0.988	*l* = −16→16
4836 measured reflections	

### Refinement

**Table:**

Refinement on *F*^2^	Primary atom site location: structure-invariant direct methods
Least-squares matrix: full	Secondary atom site location: difference Fourier map
*R*[*F*^2^ > 2σ(*F*^2^)] = 0.037	Hydrogen site location: inferred from neighbouring sites
*wR*(*F*^2^) = 0.098	H-atom parameters constrained
*S* = 1.07	*w* = 1/[σ^2^(*F*_o_^2^) + (0.0415*P*)^2^ + 0.146*P*] where *P* = (*F*_o_^2^ + 2*F*_c_^2^)/3
1166 reflections	(Δ/σ)_max_ < 0.001
73 parameters	Δρ_max_ = 0.24 e Å^−^^3^
0 restraints	Δρ_min_ = −0.16 e Å^−^^3^

### Special details

**Table:**

Geometry. All e.s.d.'s (except the e.s.d. in the dihedral angle between two l.s. planes) are estimated using the full covariance matrix. The cell e.s.d.'s are taken into account individually in the estimation of e.s.d.'s in distances, angles and torsion angles; correlations between e.s.d.'s in cell parameters are only used when they are defined by crystal symmetry. An approximate (isotropic) treatment of cell e.s.d.'s is used for estimating e.s.d.'s involving l.s. planes.
Refinement. Refinement of *F*^2^ against ALL reflections. The weighted *R*-factor *wR* and goodness of fit *S* are based on *F*^2^, conventional *R*-factors *R* are based on *F*, with *F* set to zero for negative *F*^2^. The threshold expression of *F*^2^ > σ(*F*^2^) is used only for calculating *R*-factors(gt) *etc*. and is not relevant to the choice of reflections for refinement. *R*-factors based on *F*^2^ are statistically about twice as large as those based on *F*, and *R*- factors based on ALL data will be even larger.

### Fractional atomic coordinates and isotropic or equivalent isotropic displacement parameters (Å^2^)

**Table:**

	*x*	*y*	*z*	*U*_iso_*/*U*_eq_	
O1	0.0282 (2)	−0.01262 (16)	0.31172 (9)	0.0483 (4)	
O2	0.2805 (3)	0.29340 (13)	0.49666 (9)	0.0482 (3)	
N1	0.0676 (4)	0.0393 (2)	0.14760 (11)	0.0580 (5)	
H1A	−0.0736	−0.0213	0.1141	0.070*	
H1B	0.1561	0.0889	0.1107	0.070*	
N2	0.6588 (2)	−0.04206 (14)	0.43619 (9)	0.0316 (3)	
H2C	0.7546	−0.0678	0.3948	0.038*	
C1	0.1482 (3)	0.05588 (18)	0.25545 (11)	0.0320 (3)	
C2	0.3991 (3)	0.16430 (17)	0.30689 (11)	0.0300 (3)	
H2A	0.3353	0.2749	0.3022	0.036*	
H2B	0.5274	0.1554	0.2656	0.036*	
C3	0.5568 (3)	0.12212 (17)	0.42763 (11)	0.0289 (3)	
H3A	0.7224	0.1923	0.4531	0.035*	
C4	0.3792 (3)	0.15646 (18)	0.49910 (11)	0.0307 (3)	

### Atomic displacement parameters (Å^2^)

**Table:**

	*U*^11^	*U*^22^	*U*^33^	*U*^12^	*U*^13^	*U*^23^
O1	0.0425 (6)	0.0752 (9)	0.0326 (6)	−0.0194 (6)	0.0199 (5)	−0.0009 (5)
O2	0.0730 (8)	0.0412 (6)	0.0366 (6)	0.0250 (6)	0.0267 (6)	0.0066 (5)
N1	0.0743 (11)	0.0739 (11)	0.0270 (7)	−0.0402 (9)	0.0185 (7)	−0.0063 (7)
N2	0.0336 (6)	0.0399 (7)	0.0266 (6)	0.0094 (5)	0.0172 (5)	0.0028 (5)
C1	0.0331 (7)	0.0392 (8)	0.0267 (7)	0.0000 (6)	0.0140 (6)	0.0016 (6)
C2	0.0347 (7)	0.0327 (7)	0.0261 (7)	−0.0005 (6)	0.0150 (6)	0.0024 (6)
C3	0.0292 (7)	0.0322 (7)	0.0268 (7)	−0.0005 (6)	0.0116 (6)	−0.0003 (6)
C4	0.0329 (7)	0.0364 (7)	0.0227 (7)	0.0067 (6)	0.0093 (6)	0.0003 (6)

### Geometric parameters (Å, °)

**Table:**

O1—C1	1.2304 (17)	C1—C2	1.512 (2)
O2—C4	1.2392 (17)	C2—C3	1.5309 (19)
N1—C1	1.3182 (19)	C2—H2A	0.9700
N1—H1A	0.8599	C2—H2B	0.9700
N1—H1B	0.8599	C3—C4	1.5135 (19)
N2—C4^i^	1.3219 (18)	C3—H3A	0.9800
N2—C3	1.4502 (18)	C4—N2^i^	1.3219 (18)
N2—H2C	0.8599		
			
C1—N1—H1A	119.9	C1—C2—H2B	109.1
C1—N1—H1B	120.1	C3—C2—H2B	109.1
H1A—N1—H1B	120.0	H2A—C2—H2B	107.9
C4^i^—N2—C3	127.05 (12)	N2—C3—C4	113.51 (11)
C4^i^—N2—H2C	116.4	N2—C3—C2	110.01 (11)
C3—N2—H2C	116.5	C4—C3—C2	111.46 (11)
O1—C1—N1	122.47 (14)	N2—C3—H3A	107.2
O1—C1—C2	121.49 (13)	C4—C3—H3A	107.2
N1—C1—C2	116.05 (13)	C2—C3—H3A	107.2
C1—C2—C3	112.30 (12)	O2—C4—N2^i^	122.34 (13)
C1—C2—H2A	109.1	O2—C4—C3	118.24 (13)
C3—C2—H2A	109.1	N2^i^—C4—C3	119.39 (12)

Symmetry codes: (i) −*x*+1, −*y*, −*z*+1.

### Hydrogen-bond geometry (Å, °)

**Table:**

*D*—H···*A*	*D*—H	H···*A*	*D*···*A*	*D*—H···*A*
N1—H1A···O2^ii^	0.86	2.12	2.9185 (19)	154.
N1—H1B···O2^iii^	0.86	2.03	2.8795 (18)	167.
N2—H2C···O1^iv^	0.86	2.06	2.8509 (17)	152.

Symmetry codes: (ii) −*x*, *y*−1/2, −*z*+1/2; (iii) *x*, −*y*+1/2, *z*−1/2; (iv) *x*+1, *y*, *z*.
